# Determinants of loss to follow-up among people living with HIV receiving antiretroviral therapy in a universal test and treat setting: A retrospective cohort study in Nepal

**DOI:** 10.1016/j.puhip.2025.100634

**Published:** 2025-06-26

**Authors:** Archana Shrestha, Lisasha Poudel, Bikram Adhikari, Saroj Bhandari, Roman Shrestha, Rajya Shree Kunwar, Lok Raj Pandey, Man Bahadur KC, Erin C. Wilson, Keshab Deuba

**Affiliations:** a Department of Public Health and Community Programs, Kathmandu University School of Medical Sciences, Dhulikhel, Nepal; b Center for Methods in Implementation and Prevention Science, Yale School of Public Health, New Haven, CT, USA; c Institute for Implementation Science and Health, Kathmandu, Nepal; d Save the Children International, Kathmandu, Nepal; e Department of Allied Health Sciences, University of Connecticut, Storrs, USA; f National Centre for AIDS & STD Control, Ministry of Health and Population, Kathmandu, Nepal; g San Francisco Department of Public Health, San Francisco, CA, USA; h Public Health and Environment Research Centre (PERC), Lalitpur, Nepal; i Department of Global Public Health, Karolinska Institutet, Stockholm, Sweden; j Centre for International Health (CIH), Department of Global Public Health and Primary Care, University of Bergen, Bergen, Norway

**Keywords:** Test and treat, Antiretroviral therapy, People living with HIV, Nepal

## Abstract

**Objectives:**

This study aims to assess the cumulative incidence and rate of loss to follow-up (LTFU) among people living with HIV (PLHIV) in Nepal who begin antiretroviral therapy (ART) early, as well as to identify factors associated with LTFU in the context of the universal test and treat approach.

**Study design:**

Retrospective cohort study.

**Methods:**

We retrospectively analysed nationally representative routine programme data for all PLHIV initiated on ART from February 19, 2017, to February 18, 2020, and followed up until May 10, 2022. LTFU was defined as a client not returning to the HIV clinic for at least 3 months from the date of their last scheduled appointment. We calculated cumulative incidence rate (IR) and used a multivariable Cox proportional hazards regression model to identify factors associated with LTFU, reporting corresponding 95% confidence intervals (CI).

**Results:**

Of the 8192 clients included in our sample, 6797 (82.9 %) started ART within seven days following their HIV diagnosis. The overall IR of LTFU was 4.22 (95 % CI = 3.95–4.51) per 100 person years of observation. The cumulative incidence of LTFU increased over time on ART, from 3.81 % (95 % CI = 3.40–4.26) at 6 months, 6.51 % (95 % CI = 5.97–7.09) at 12 months to 13.41 % (95 % CI = 12.51–14.37) at 48 months. In the multivariable model, factors associated with higher odds of LTFU included initiating treatment within 7 days of diagnosis, younger age, being unmarried, belonging to the Dalit caste, having WHO clinical stage 1 at baseline, and initiation on a non-nucleoside reverse transcriptase inhibitors (NNRTI)-based regimen. Among key populations, sex workers, their clients, men who have sex with men and transgender, people who inject drugs were at higher risk of dropout compared to migrants.

**Conclusions:**

In this nationwide cohort, the risk of LTFU increased with time on ART. To optimize the test-and-treat strategy in Nepal, it is crucial to address the unique needs of youth and certain key populations and manage any early adverse drug reactions.

## 1 Introduction

HIV continues to threaten public health, with an estimated 39.9 million people living with HIV (PLHIV), 1.3 million new infections, and 630,000 deaths globally in 2023 [1]. The United Nations Joint Programme on HIV/AIDS (UNAIDS) Global AIDS Strategy 2021–2026 set ambitious targets and commitments for 2025, known as the 95-95-95 goals, to accelerate the HIV epidemic control. These targets aim to ensure that: (a) 95 % of PLHIV will know their status; (b) 95 % of PLHIV who know their HIV status are on antiretroviral therapy (ART); and (c) 95 % of PLHIV on ART have suppressed viral load, by 2025 [2]. The timing of ART initiation determines risks for onward HIV transmission and the prognosis of the person who tests positive. Initiation of ART immediately after a positive HIV test result reduces HIV transmission and AIDS-related complications [[3], [4], [5], [6]]. These findings prompted the WHO to launch the ‘treat-all’ guidelines, known as the test-and-treat strategy, which recommended initiating ART regardless of CD4 count or clinical stage in 2015. [7]. In 2017, the WHO further recommended rapid ART initiation, i.e., starting ART as soon as possible after HIV diagnosis, ideally on the same day for those ready to start or within 7 days of diagnosis [8]. Evidence has shown that the test-and-treat strategy, through early initiation of treatment and adherence support, impacts all epidemiological aspects of HIV and AIDS. Notably, it leads to longer survival, better immune reconstitution, and lower mortality rates [[9], [10], [11]].

In February 2017, the Nepal government launched the test-and-treat strategy and implemented it across all 68 then-existing ART sites nationwide [12]. This has dramatically increased ART initiation among PLHIV [13]. Concerns have been raised regarding the capacity of healthcare systems to accommodate the increasing number of new PLHIV, as well as the availability of ART medications due to historical supply chain issues and limitations in viral load testing capacity, including reagents and cartridges [14]. Additionally, the already limited availability of HIV test kits for diagnosing and confirming new positive cases has been highlighted [15]. Ethical concerns have been raised, particularly regarding potential coercion, where providers may feel pressured to encourage PLHIV to initiate ART at diagnosis [16]. Additionally, the test-and-treat strategy may increase the risk of drug resistance in PLHIV who start ART early but later struggle with adherence. Concerns regarding resistance are compounded by the limited treatment options within various drug classes, especially during the transition from first-line to second-line therapy in Nepal. Furthermore, significant lost-to-follow-up was observed in trials of rapid ART initiation [17].

Currently, there are 85 HIV treatment clinics in 61 districts in Nepal that provide free ART to all PLHIV. Additionally, there are 45 antiretroviral dispensing sites to improve accessibility for PLHIV, covering 76 districts of Nepal [18]. The national treatment cascade in 2023 showed that 94 % of the estimated 30,300 PLHIV in Nepal were aware of their HIV status. Among these individuals, 87 % (n = 24,793) were receiving ART; of those, 76 % (n = 18,805) underwent viral load testing in 2023, and 74 % (n = 18,236) achieved viral suppression [18]. Retention across all age groups on ART is 87 % at 12 months, 85 % at 24 months and 84 % at 36 months [18].

In settings where clients start ART immediately following a positive HIV test result, many may not return to the HIV care after initiating, which offsets the advantages of rapid ART starts. Data from PLHIV with rapid ART initiation indicates suboptimal ART adherence and retention, with the majority being loss-to-follow-up (LTFU) [19,20]. HIV treatment centers may find it difficult to meet the existing demand for regular care, bringing into question whether high retention is possible within the universal test-and-treat approach. Known LTFU factors before the test-and-treat era were young age, male sex, low education level, occupation, and mobility [[21], [22], [23], [24], [25]] at the individual level, and disengagement in care, long wait times, negative attitude of caregivers, and the necessity for frequent visits at the system level [[26], [27], [28]]. These factors may still affect retention in the test-and-treat era. New studies are needed to understand the gaps around retention in care within routine health care delivery settings. In the given context, we aimed to estimate the cumulative incidence, incidence rate of loss to follow up, and factors associated with loss to follow up in ART clinics that began implementing the test-and-treat strategy in 2017 within routine health care settings in Nepal.

## 2 Methods

### 2.1 Study design

This was a retrospective cohort study using data of clients diagnosed with HIV and enrolled at 80 ART clinics from 61 districts in Nepal from February 19, 2017 to February 18, 2020. A client's ART initiation date was set as the beginning of follow-up (time zero), and the follow-up period ended on May 10, 2022. The clients' follow-up ended if they died, were LTFU or were censored.

### 2.2 Study site and settings

In Nepal, ART services started in February 2004 at the Sukraraj Tropical and Infectious Disease Hospital in the Kathmandu district, the capital city. By 2022, ART services had been had been scaled up to cover the entire country. PLHIV in Nepal have access to ART free of charge. Nepal has also committed to achieving the Sustainable Development Goals (SDG), including the target of ending the AIDS epidemic by 2030. The National HIV Strategic Plan 2021-2026 has endorsed the global commitment of 95-95-95 targets by 2026 [29]. In 2017, the National HIV programme implemented the test-and-treat strategy that provided ART to all PLHIV regardless of the CD4 counts or WHO stage in Nepal. Before that, only PLHIV with a CD4 cell count of less than 500 cells per microliter (μL), WHO clinical stages 3 or 4, pregnant or breastfeeding mothers were eligible for ART. Diverse models of HIV testing services (HTS) are available in Nepal to increase access to HIV diagnosis, including testing services in healthcare facilities and at stand-alone sites, as well as through a range of community-based approaches.

In Nepal, all individuals diagnosed with HIV receive standardized post-test counseling as part of the national HIV care and treatment program. This counseling includes comprehensive information on the nature of HIV infection and its progression in the absence of treatment. Clients are educated on adopting positive prevention behaviors to maintain their health and minimize the risk of onward transmission. The benefits of ART are emphasized, particularly its role in preventing disease progression to AIDS and in reducing HIV transmission risk. Counselors provide detailed explanations of viral load suppression and its significance, including the “Undetectable = Untransmittable” (U=U) principle, which conveys that individuals with a sustained undetectable viral load do not transmit HIV to others. Clients are also encouraged to initiate ART promptly, attend regular follow-up visits, adhere to prescribed treatment, and manage other health conditions, including opportunistic infections. The counseling aims to promote long-term retention in care and optimize clinical outcomes through sustained treatment adherence.

### 2.3 Study participants

We included PLHIV aged 18 years and above registered in the national HIV programme with a ‘take all approach’ as per the availability of the data on the national electronic register [HIV care and ART tracking (DHIS2 Tracker, mHealth and Biometric) system]. The inclusion criteria were PLHIV enrolled in ART services and recorded ART outcomes (LTFU, Death, Transfer out, Alive).

### 2.4 Data collection and management

Co-authors working within the national HIV programme extracted relevant electronic data from the national database according to standard guidelines for handling individual-level data, ensuring the exclusion of personal identifiers [30]. The research team performed pre-analytical processing including data cleaning, labelling and checking missingness using an R version 4.2.8. The programme staff conducted active defaulter tracing and data auditing comparing paper-based and electronic datasets, ensuring reliability of data, specifically outcome LTFU. The abstracted data were checked for completeness and accuracy of dates of ART initiation, medical history-filled, HIV positive status, treatment substituted (changing one drug within the existing regimen, typically due to side effects or adherence issues), treatment switched (changing the entire ART regimen due to treatment failure or resistance to regain viral control), and date of the end of the follow up period if any of these dates were recorded after the date of registration. The list of missing and erroneous variables in the client-wise list, with the unique client identifier and serial number were handed to the respective ART sites that were cross-checked against the source paper-based recording registers used at ART centers and entered by health workers into the electronic system. An anonymized file was included in this review, meaning it did not contain any personal identifiers of PLHIV, such as names or mobile numbers. The unique client code facilitated communication between the team and ART clinics. This process was iteratively used in all ART sites.

### 2.5 Variables

#### 2.5.1 Outcome variable

The primary outcome variable was LTFU. LTFU was defined as a person who missed their last scheduled appointment by at least three months or was classified as ‘missing’ for less than three months from the date of their last scheduled appointment, without being documented as deceased or officially transferred out [31].

#### 2.5.2 Independent variables

The independent variables included sociodemographic characteristics, medical history, ART related information, and HIV care history. The ***socio-demographic characteristics*** comprised of age (in years), sex (male, female, others or third gender), ethnicity and caste (Brahmin/Chhetri, Dalit, Janajati, Madhesi, Muslim, Others), marital status (married, living together, unmarried, widow/widower, divorced), monthly income (in Nepalese Rupees), educational status (illiterate, primary, secondary, higher secondary, bachelor and above), and province (Koshi, Madhesh, Bagmati, Gandaki, Lumbini, Karnali, Sudurpashchim). The risk group classification included client of sex workers, migrants, men who have sex with men (MSM), transgender (TG), people who inject drugs (PWID), sex workers, spouses of migrants, transgender individuals, those with vertical transmission, and other groups such as blood/organ transplant recipients and prison inmates. The ***medical history*** encompassed a history of tuberculosis (yes, no), the presence of chronic disease (yes, no), and the presence of opportunistic infections (yes, no). ART-related information included the ART start date and the ART regimen (Integrase Strand Transfer Inhibitor (INSTI), Non-nucleoside Reverse Transcriptase Inhibitors (NNRTI), Protease Inhibitor (PI), Not Applicable). **HIV care history** was documented by the date of HIV positive diagnosis, the date of ART enrollment, baseline WHO clinical stage (stage 1, stage 2, stage 3, stage 4), baseline CD4 count (in cells/μL), and follow-up CD4 count (in cells/μL).

The study team did not present descriptive statistics for two variables: baseline CD4 count (in cells/μL) and follow-up CD4 count (in cells/μL). This was due to the low number of PLHIV tested for CD4 after the implementation of the test-and-treat strategy, even though the guidelines do not recommend against using CD4 tests to monitor immunological status. The available CD4 results are primarily from PLHIV, who presented late for HIV care. Presenting these results would significantly underestimate the mean or median CD4 values.

### 2.6 Statistical methods

We summarized the clients’ characteristics using mean and standard deviation (SD) for parametric numerical variables, median and interquartile range (IQR) for non-parametric numerical variables, and frequency and percentage for categorical variables. We described the longitudinal change in LTFU)/missing using the Kaplan-Meier plot. We calculated the duration of LTFU by subtracting clients’ enrollment date from their last date of visit. The factors associated (independent variables) with optimal LTFU/missing and stopped treatment were assessed using the cox-proportional hazard model. We analysed data at bivariate level to estimate predictors and multivariable Cox regression model to estimate adjusted predictors of time to LTFU. The multivariable model was built using a stepwise approach. We included variables with a p value of less than 0.2 in bivariate and dropped each turning out with a p > 0.05 at multivariable model. Insignificant at this stage but considered significantly associated with LTFU in previous literature were included in the final model. We presented the result of multivariable cox regression model as crude and adjusted hazard ratio (aHR) and its 95 % confidence interval (CI). We evaluated proportional hazards assumptions for each variable included in the final model.

### 2.7 Ethical consideration

We received ethical approval from the ethical review board (ERB) at the Nepal Health Research Council (Reference number: 97, Date of approval: 2021-07-25). The study was retrospective and hence the client's consent could not be obtained, this was acknowledged in our ERB application. Routinely collected programme data were extracted from e-registers without the client's direct identifiers. All data was kept in a password-protected computer.

## 3 Results

**Fig. 1:**
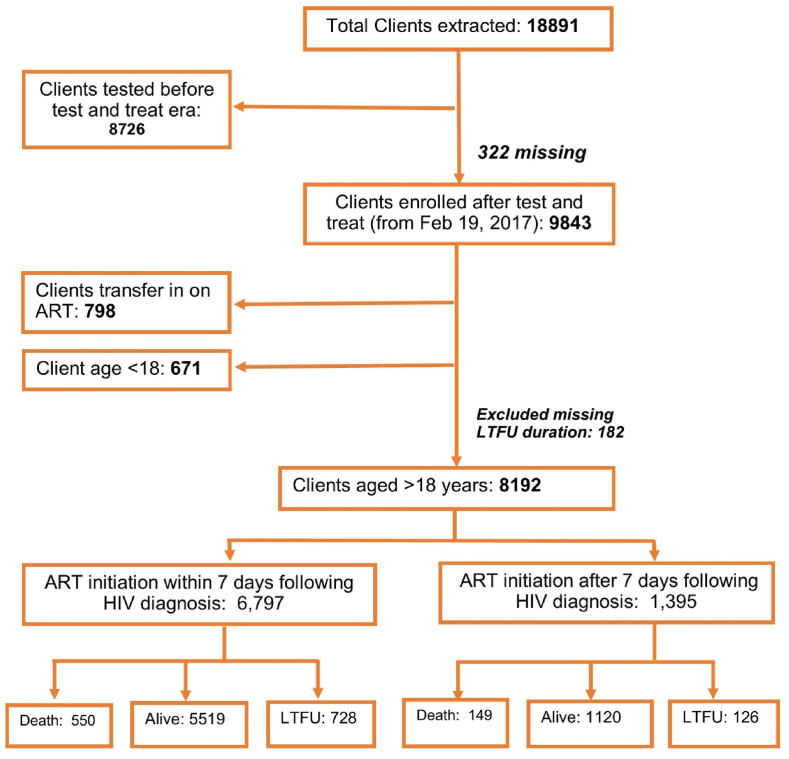
A flow diagram showing clients abstraction and inclusion into the study and their outcomes.

We extracted data for 18,891 clients, and 8192 clients met the study inclusion criteria (Fig. 1).

### 3.1 Characteristics of study participants

Details of the baseline clients’ characteristics are presented in Table 1. The mean age at ART initiation was 36.6 (SD = 10.9) years. There were more males (56 %) than females. Janajati was the predominant ethnic group (30.9 %).

**Table 1:** Clients background characteristics. (n = 8192).

Characteristics	Overall n (%)	ART initiated within 7 days	ART initiated after 7 days	p-value
**Age at registration, in years, mean (SD)**	**36.6 (10.9)**	**36.5(11.0)**	**37.1(10.7)**	**0.06**
**Sex**				
Male	4585 (56.0)	3763 (55.4)	822 (58.9)	0.05
Female	3417 (41.7)	2873 (42.3)	544 (39.0)	
Others (Third gender)	190 (2.3)	161 (2.4)	29 (2.0)	
**Ethnicity**				
Brahmin/Chhetri	2291 (28.0)	1945 (28.6)	346 (24.8)	<0.01
Dalit	1433 (17.5)	1193 (17.5)	240 (17.2)	
Janajati	2531 (30.9)	2123 (31.2)	408 (29.2)	
Madhesi	1405 (17.2)	1098 (16.1)	307(22.0)	
Muslim	310 (3.8)	257 (3.8)	53 (3.8)	
Others	222 (2.7)	181 (2.6)	41 (2.9)	
**Marital status**				
Married	6057 (74.2)	5012 (74.0)	1045 (74.9)	0.04
Unmarried	1063 (13.0)	902 (13.3)	161 (11.5)	
Widow/Widower	816 (10.0)	658 (9.7)	158 (11.3)	
Divorced	230 (2.8)	199 (2.9)	31 (2.2)	
**Monthly income, in NPR mean (SD)**	5696.1 (11825.7)	5631.3 (12057.7)	6002.0 (10660.6)	0.30
**Education**				
Illiterate	3749 (45.8)	3091 (45.5)	658 (47.2)	0.01
Primary	2055 (25.1)	1688 (24.8)	367 (26.3)	
Secondary	1374 (16.8)	1166 (17.1)	208(14.9)	
Higher Secondary	517 (6.3)	417 (6.1)	100 (7.2)	
Bachelor and above	88 (1.1)	77(1.1)	11(0.8)	
Unknown	409 (5.0)	358 (5.3)	51 (3.6)	
**Baseline WHO clinical stage**				
Stage-1	4728 (58.1)	4056(60.0)	672(48.4)	<0.01
Stage-2	1890 (23.1)	1554 (23.0)	336 (24.2)	
Stage-3	1181 (14.5)	912 (13.5)	269 (19.4)	
Stage-4	345 (4.2)	235 (3.5)	110 (7.9)	
**History of Tuberculosis**				
Yes	456 (5.6)	318 (4.7)	138 (9.9)	<0.01
No	7722(94.4)	6467 (95.3)	1255 (90.1)	
**Presence of Chronic Disease**				
Yes	103 (1.3)	78 (1.1)	25 (1.8)	0.05
No	8089 (98.7)	6719 (98.8)	1370 (98.2)	
**Presence of Opportunistic Infection**^a^				
Yes	363 (4.4)	256 (3.8)	107 (7.7)	<0.01
No	7817 (95.6)	6530 (96.2)	1287 (92.3)	
**ART regimen**				
INSTI	5898 (72.0)	5005 (73.6)	893 (64.0)	<0.01
NNRTI	2155 (26.3)	1679 (24.7)	476 (34.1)	
PI	69 (0.8)	52 (0.8)	17 (1.2)	
Not available (missing or other regimen)	70 (0.8)	61 (0.9)	9 (0.6)	
**Risk group**				
Client of Sex worker	1215(14.8)	1000 (14.7)	215(15.4)	<0.01
Migrants	2484 (30.3)	1999 (29.4)	485 (34.8)	
MSM	461 (5.6)	403 (5.9)	58 (4.2)	
PWID	292 (3.6)	247 (3.6)	45 (3.2)	
Sex worker	297(3.6)	246 (3.6)	51 (3.7)	
Spouse of migrants	2505 (30.6)	2104 (30.9)	401 (28.7)	
Transgender	35 (0.4)	30 (0.4)	5 (0.4)	
Other risk groups^b^	903 (11.0)	768 (11.3)	135 (9.7)	
**Province**				
Koshi	917 (11.2)	786 (11.6)	131 (9.4)	<0.01
Madhesh	1400 (17.1)	1057 (15.6)	343 (24.6)	
Bagmati	2195 (26.8)	1838 (27.0)	357 (25.6)	
Gandaki	871 (10.6)	799 (11.67	72 (5.2)	
Lumbini	1620 (19.8)	1346 (19.8)	274 (19.6)	
Karnali	192 (2.3)	167 (2.5)	25 (1.8)	
Sudurpashchim	994 (12.1)	801 (11.8)	193 (13.8)	
**Status**				
Alive	7452 (91.9)	6213 (92.3)	1239 (89.9)	<0.01
Death	660 (8.1)	521 (7.7)	139 (10.1)	

**n: frequency; %: percentage; SD:** Standard Deviation**; NPR:** Nepalese Rupee**; INSTI:** Integrase Strand Transfer Inhibitor**; NNRTI:** Non-nucleoside reverse transcriptase inhibitors**; PI:** Protease inhibitor.

a **Opportunistic Infection:** Bacterial pneumonia, Candidiasis, Crypto Meningitis, Diarrhea, Genital Herpes, Herpes Zoster, Pneumocystis pneumonia, Toxoplasmosis, Tuberculosis, and Others.

b **Other risk group** includes blood organ transplant, prison inmates, vertical transmission, other).

The majority of clients were married (74.2 %), many were illiterate (45.8 %), migrants (30.3 %), and most started ART in WHO clinical stage 1 (58.1 %), started treatment with INSTI (72.0 %), had no history of Tuberculosis (94.4 %) or chronic disease (98.7 %) or currently had an opportunistic infection (95.6 %). Overall, 91.9 % of clients were alive, while 8.1 % were reported as deceased.

Among PLHIV who initiated ART more than seven days after diagnosis, the median time to ART initiation was 20 days. Of these individuals, 54.3 % commenced ART within 1–2 weeks, 21.0 % started between 2 and 4 weeks, and 15.3 % within 1–3 months. A smaller but significant proportion (9.5 %) initiated ART more than three months after diagnosis (data not shown in the table).

**Fig. 2:**
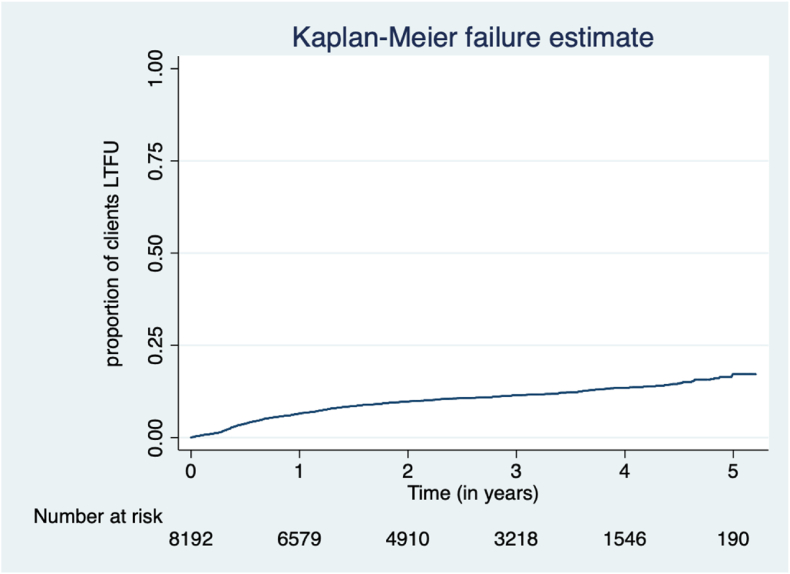
Overall clients lost to follow up in the study period.

**Fig. 3:**
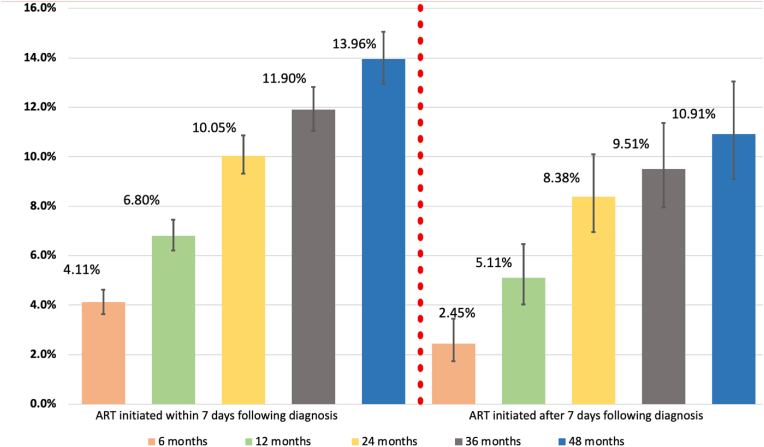
Cumulative incidence of loss to follow up by study group at different time points.

There were **854** cases of LTFU observed over **20,235.94** person-years at risk. The overall incidence rate (IR) of LTFU was 4.22 (95 % CI = 3.95–4.51) per 100 person-years of observation (Pyo). Cumulative incidence of LTFU was 3.81 % (95 % CI = 3.40–4.26) at 6 months, 6.51 % (95 % CI = 5.97–7.09) at 12 months, 9.77 % (95 % CI = 9.09–10.49) at 24 months, 11.47 % (95 % CI = 10.72–12.28) at 36 months and 13.41 % (95 % CI = 12.51–14.37) at 48 months. Fig. 2 shows the overall cumulative incidence of LTFU during the study period. Fig. 3 illustrates the proportion of clients LTFU by study group.

### 3.2 Incidence rate of loss to follow up by clients’ characteristics

Table 2 presents incidence rates (IR) of LTFU by clients' baseline characteristics. The incidence rate of LTFU was higher (4.42 versus 3.34) in the group that started ART within 7 days following diagnosis compared to the group that started ART after 7 days following diagnosis. The incidence rate of LTFU was higher in clients who self-reported as a third gender, aged 18–24 years old, were unmarried, of Janajati ethnicity, and were sex workers. The incidence rate of LTFU was higher among clients with fourth stage HIV at baseline, with CD4 count greater than 351, and those on NNRTI. Clients residing in Bagmati and Karnali provinces had a higher incidence rate of LTFU.

**Table 2:** Incidence rate of loss to follow up by clients’ characteristics.

Characteristics	No. of cases	Person time	Rate/100 PYO	95 % CI
**Study Group**	**854**	**20235.94**	**4.22**	**3.95–4.51**
**ART initiation**				
After 7 days	126	3776.23	3.34	2.80–3.97
Within 7 days	728	16459.71	4.42	4.11–4.76
**Sex**				
Female	292	9083.69	3.21	2.87–3.60
Male	530	10821.61	4.90	4.50–5.33
Others (Third gender)	32	330.64	9.68	6.84–13.68
**Age**				
18-24	167	2701.12	6.18	5.31–7.19
24-29	151	3005.91	5.02	4.28–5.89
30-39	287	7166.13	4.00	3.57–4.50
40-49	167	4816.97	3.47	2.98–4.03
50+	82	2545.80	3.22	2.59–4.00
**Ethnicity**				
Brahmin/Chhetri	216	5843.89	3.70	3.23–4.22
Dalit	150	3480.70	4.31	3.67–5.06
Janajati	312	6326.77	4.93	4.41–5.51
Madhesi	122	3285.2	3.71	3.11–4.43
Muslim	27	711.87	3.79	2.60–5.53
Others	27	587.51	4.59	3.15–6.70
**Marital status**				
Married	550	15153.47	3.63	3.34–3.94
Unmarried	189	2325.45	8.13	7.05–9.37
Widow/Widower	70	2148.08	3.26	2.58–4.12
Divorced	41	556.23	7.37	5.43–10.01
**Monthly income, in Nepalese Ruppee**				
<10,000	555	13701.37	4.05	3.73–4.40
10,001–30,000	194	4348.34	4.46	3.87–5.13
>30,000	8	166.70	4.80	2.40–9.60
**Education**				
Illiterate	349	9076.34	3.84	3.46–4.27
Primary	239	4824.62	4.95	4.36–5.62
Secondary	141	3404.52	4.14	3.51–4.88
Higher Secondary	50	1373.64	3.64	2.76–4.80
Bachelor and above	6	188.76	3.18	1.43–7.07
Unknown	69	1368.05	5.04	3.98–6.38
**WHO stage at the time of registration**				
Stage 1	550	11446.85	4.80	4.42–5.22
Stage 2	157	5178.96	3.03	2.59–3.54
Stage 3	98	2778.47	3.52	2.89–4.30
Stage 4	43	722.01	5.95	4.42–8.03
**History of Tuberculosis**				
Yes	48	1025.64	4.68	3.53–6.21
No	805	19179.15	4.20	3.92–4.50
**Presence of Chronic Disease**				
Yes	5	259.92	1.92	0.80–4.62
No	849	19976.02	4.25	3.97–4.54
**Presence of opportunistic infections**				
Yes	33	19455.16	4.41	3.14–6.21
No	820	747.71	4.21	3.93–4.51
**Baseline CD4 count**				
<200	56	2326.36	2.41	1.85–3.13
200-350	44	1661.04	2.65	1.97–3.56
>351	84	2880.74	2.91	2.35–3.61
**ART regimen**				
INSTI	279	16246.23	1.72	1.53–1.93
NNRTI	566	3598.45	15.73	14.48–17.08
PI	5	230.21	2.17	0.90–5.22
Not Applicable (missing or other regimen)	4	161.06	2.48	0.93–6.62
**Risk group**				
Client Sex worker	184	2927.4	6.28	5.44–7.26
Migrants	206	5765.05	3.57	3.12–4.10
MSM	68	849.45	8.00	6.31–10.15
PWID	65	760.72	8.54	6.70–10.89
Sex worker	62	680.69	9.12	7.10–11.68
Spouse of migrant	157	6770.69	2.32	1.98–2.71
Transgender	4	59.66	6.70	2.52–17.86
Other risk groups^a^	108	2422.72	4.46	3.70–5.34
**Province**				
Koshi	107	2367.81	4.52	3.74–5.46
Madhesh	103	3143.53	3.28	2.70–3.97
Bagmati	355	5719.31	6.21	5.59–6.89
Gandaki	93	2235.83	4.16	3.39–5.10
Lumbini	132	3919.04	3.37	2.84–3.99
Karnali	25	478.69	5.22	3.53–7.73
Sudurpashchim	39	2363.68	1.65	1.20–2.26

a Other risk group includes blood organ transplant, prison inmates, vertical transmission, do not want to disclose.

### 3.3 Factors associated with loss to follow up

Table 3 presents the factors associated with LTFU from national register data during the test-and-treat all era. The risk of LTFU was 48 % higher in clients who started ART within 7 days of HIV diagnosis compared to those who initiated ART after seven days (aHR: 1.48, 95 % CI, 1.22–1.80). Age above 40 years was significantly associated with lower risk of LTFU (aHR: 0.76, 95 % CI: 0.6–0.96) compared to PLHIV aged 18–24 years. Dalit caste was significantly associated with a higher risk of LTFU compared to Brahmin/Chhetri (aHR: 1.24, 95 % CI: 1.00–1.55, p = 0.05). Unmarried PLHIV had a significantly higher risk of LTFU compared to married individuals (aHR: 1.29, 95 % CI: 1.05–1.58, p = 0.02). Higher education was significantly associated with lower LTFU (p-trend = <0.01) compared to PLHIV with no formal education.

**Table 3:** Factors associated with loss to follow up/missing in the test-and-treat era period in Nepal (n = 8119).

Characteristics	Univariate				Multivariable			
	Crude HR	95 % CI		p-value	aHR	95 % CI		p-value
**ART initiation**								
ART initiated after 7 days	Ref							
ART initiated within 7 days	1.28	1.06	1.55	0.01	1.48	1.22	1.80	<0.01
**Sex**								
Female	Ref							
Male	1.48	1.28	1.71	<0.01	0.94	0.73	1.21	0.62
Others (Third gender)	2.62	1.82	3.78	<0.01	1.35	0.82	2.22	0.24
**Age**								
118-24 years	Ref							
225-29 years	0.83	0.67	1.04	0.11	0.97	0.77	1.23	0.83
30–39 years	0.67	0.55	0.81	<0.01	0.88	0.70	1.09	0.20
>40 years	0.55	0.45	0.68	<0.01	0.76	0.60	0.96	0.02
**Ethnicity**								
Brahmin/Chhetri	Ref							
Dalit	1.15	0.94	1.42	0.17	1.24	1.00	1.55	0.05
Janajati	1.32	1.11	1.57	<0.01	1.04	0.86	1.24	0.70
Madhesi	0.99	0.79	1.24	0.94	1.01	0.78	1.32	0.93
Muslim	0.10	0.67	1.49	0.99	1.21	0.79	1.84	0.38
Others	1.27	0.85	1.89	0.25	0.92	0.61	1.38	0.69
**Marital Status**								
Married	Ref							
Divorced	2.00	1.45	2.74	<0.01	1.31	0.94	1.82	0.16
Unmarried	2.12	1.79	2.50	<0.01	1.29	1.05	1.58	0.02
Widow/Widower	0.91	0.71	1.17	0.47	1.12	0.86	1.46	0.39
**Education**								
Illiterate	Ref							
Primary	1.26	1.07	1.49	<0.01	0.98	0.82	1.16	0.78
Secondary	1.07	0.88	1.30	0.51	0.77	0.62	0.95	0.01
Higher secondary	0.96	0.71	1.29	0.79	0.58	0.42	0.79	<0.01
Bachelor and above	0.76	0.34	1.70	0.50	0.5	0.22	1.13	0.09
Unknown	1.45	1.12	1.87	<0.01	0.71	0.53	0.94	0.02
**WHO stage at the time of registration**								
Stage 1	Ref							
Stage 2	0.66	0.55	0.79	<0.01	0.76	0.63	0.91	<0.01
Stage 3	0.76	0.61	0.94	0.01	0.7	0.56	0.88	<0.01
Stage 4	1.24	0.91	1.70	0.16	0.97	0.7	1.34	0.86
**History of Chronic Disease**								
No	Ref							
Yes	0.46	0.19	1.10	0.08	0.39	0.14	1.04	0.06
**Types of ART**								
INSTI	Ref							
NNRTI	8.67	7.51	10.01	<0.01	11.51	9.89	13.4	<0.01
PI	1.34	0.55	3.26	0.51	1.65	0.68	4	0.27
NA	1.43	0.53	3.84	0.47	1.62	0.6	4.36	0.34
**Risk group**								
Migrants	Ref							
Sex worker	2.51	1.89	3.34	<0.01	1.58	1.09	2.27	0.01
Client sex worker	1.75	1.44	2.14	<0.01	1.38	1.11	1.72	<0.01
MSM and TG	2.02	1.53	2.65	<0.01	1.44	1.02	2.02	0.04
Other risk groups (blood organ transplant, prison inmates, vertical transmission, do not want to disclose)	1.29	1.02	1.63	0.03	1.01	0.77	1.34	0.92
PWID	2.46	1.86	3.25	<0.01	1.49	1.1	2.01	<0.01
Spouse of migrants	0.67	0.54	0.82	<0.01	0.62	0.45	0.84	<0.01
Transgender	1.66	0.62	4.48	0.31	1.47	0.49	4.38	0.49
**Province**								
Bagmati	Ref							
Koshi	0.73	0.59	0.91	<0.01	0.98	0.78	1.23	0.86
Madhesh	0.51	0.41	0.63	<0.01	0.27	0.2	0.36	<0.01
Gandaki	0.67	0.53	0.84	<0.01	1.02	0.79	1.31	0.87
Lumbini	0.53	0.44	0.65	<0.01	0.59	0.47	0.74	<0.01
Karnali	0.85	0.56	1.27	0.418	1.15	0.75	1.78	0.52
Sudurpaschim	0.26	0.19	0.36	<0.01	0.31	0.22	0.45	<0.01

Ref: reference group; HR: hazard ratio; aHR: adjusted hazard ratio; CI: confidence interval.

Compared to those who enrolled at the WHO clinical stage 1, the risk of LTFU was lower among clients with stage 2 (aHR: 0.76; 95 % CI:0.63–0.92) and stage 3 (aHR: 0.70; 95 % CI: 0.56–0.88). Clients taking ART regimens based on NNRTI were 11.5 times more likely to have LTFU (aHR:11.50; 95 % CI:9.88–13.39).

Compared to migrant workers, sex workers had 1.5 times higher LTFU incidence (aHR:1.55; 95 % CI: 1.07–2.24); clients of sex workers were 1.4 times higher LTFU incidence (aHR: 1.38; 95 % CI: 1.11–1.71), MSM and TG had 1.4 times higher LTFU incidence (aHR: 1.42; 95 % CI: 1,01–1.99), PWID had 1.4 times higher LTFU incidence (aHR: 1.48; 95 % CI:1.09–2.00); and spouse of the migrant worker had 0.6 times lower LTFU incidence (aHR: 0.61; 95 % CI: 0.45–0.83). Compared to Koshi Province, PLHIV had 83 % lower LTFU risk in Madhesh province (aHR: 0.27; 95 % CI: 0.20–0.37), 40 % lower LTFU risk in Lumbini (aHR: 0.60; 95 % CI: 0.46–0.79) and 79 % lower LTFU risk in Sudurpashchim (aHR: 0.31; 95 % CI: 0.21–0.47).

## 4 Discussion

In our retrospective observational study of comprehensive data on all publicly funded care clients in the national register for PLHIV in Nepal, the LTFU incidence rate was 4.22 per 100 person-years of follow up. The cumulative LTFU incidence rate increased steadily over the course of implementation of the test-and-treat strategy, reaching 13.4 % over a 48-month period. Risk factors for high LTFU among PLHIV included initiating treatment within 7 days of diagnosis, younger age, being unmarried, low socio-economic status, WHO stage 1 at baseline, being on NNRTI treatment, and residing in Koshi Province. Within risk groups, sex workers, their clients, PWID, MSM, and TG were at a higher risk of dropout compared to seasonal labor migrants. In this study, gender and a history of chronic disease were not associated with LTFU.

We observed a consistently increasing cumulative incidence of LTFU among PLHIV in Nepal during the test-and-treat implementation era. At 48 months, 89 % of the clients were retained in treatment, below the UNAIDS target of 95 % retention rate [2]. Similarly, the retention rate at 12 months was 93 %. This retention rate is higher compared to African settings with universal test-and-treat strategy such as the studies conducted in East Africa [32] and Malawi [33] where a retention rate of 89 % and 76 % at 12 months were reported during this era. Better retention rates in research from East Africa may be due to differences in the study period analysed and inclusion of the adolescent population that historically has higher LTFU rates [[32], [33], [34]].

There have been concerns regarding the possibility of achieving high HIV treatment retention and adherence with universal test-and-treat, specifically among asymptomatic PLHIV [14,35,36]. In our study, PLHIV who started ART within 7 days after diagnosis were more likely to become LTFU than those who started ART later. These findings are in line with other studies [37,38]. PLHIV who initiate early have less time to accept their HIV status and fully understand treatment options. People are more likely to retain in treatment if they understand ART [39]. Some studies have suggested that a delay in treatment among newly diagnosed PLHIV provides them necessary time to accept their HIV status [40,41] and these PLHIV may subsequently return to ART centers for care [42,43]. Additionally, PLHIV need sufficient counseling to understand about HIV status and implications of starting treatment [44].

In our study, PLHIV with the WHO stages 2 or 3 at baseline were less likely to have LTFU than those in WHO stage 1. Our findings contrast those of previous studies, which suggested that advanced HIV disease is linked with a lower chance of LTFU. We performed our analysis including those recruited after implementation of the universal test-and-treat strategy, leading to recruiting PLHIV early on without symptoms. Asymptomatic PLHIV are more prone to LTFU [45].

In our study, the younger age group had a higher rate of LTFU compared to older people, similar to studies from Asia [46,47] and the United States [48]. Young adults in many low resource settings have shown to have higher LTFU due to rigid ART clinic visit schedules that do not adjust for school and work and because ART services can be less youth-friendly [49,50]. Some studies also emphasized that young PLHIV face a greater risk of mental and behavioural health issues, resulting in low retention [51]. Furthermore, young people might be less employed and face financial difficulties accessing health facilities due to indirect costs like transportation, despite the Nepal government providing free ART.

The higher LTFU rate was observed among PLHIV from the Dalit community. Historically ostracized as “untouchables,” Dalits in Nepal continue to face pervasive discrimination in healthcare settings and limited access to health opportunities, despite recent legal reforms. Further exacerbating this issue, evidence in Nepal [[52], [53], [54], [55], [56]] indicate that people belonging to Dalit caste experience elevated health problems due to profound inequalities in health service access and utilization. Additionally, Dalits demonstrate the lowest knowledge regarding HIV prevention and transmission methods [57], compounding their vulnerability. These challenges are further intensified by disproportionate economic vulnerability [58] and a debilitating “double stigma” rooted in both their HIV status and caste identity. Such factors create significant practical barriers, including the financial burden of transport costs and the loss of essential daily wages due to clinic visits. Collectively, these multifaceted disadvantages severely erode adherence to treatment and retention in care for Dalit PLHIV.

Lower loss to follow-up among HIV clients was observed in Madhesh, Sudurpaschim, and Lumbini provinces compared to Bagmati. One possible explanation for this difference is that Bagmati, being a central urban hub, often serves as the initial point of diagnosis for many clients who later return to their home districts for continued care. This migration can lead to disruptions in follow-up and care continuity, thereby contributing to a higher loss to follow-up rate in Bagmati.

Low LTFU rate was seen among PLHIV with high educational attainment, which is consistent with a previous study in Asia of HIV care [46,47]. This might be due to two general pathways. First, higher education is indicative of a higher level of knowledge and in general acceptable healthy behaviour [59,60]. In previous qualitative findings also, knowledge and awareness were associated with ART continuation by both health workers and ART clients [61,62]. Low education also often leads to lower socio-economic status, which is independently associated with higher LTFU [63].

We found that marital status was associated with LTFU in Nepal, similar to other study [64]. Married PLHIV who disclose their status to their spouse, get psychological and emotional support, are regularly reminded of medication, and are taken care of when they are ill [64,65].

In our study, clients taking NNRTI were 11 times more likely to have LTFU compared to INSTI. This might be a result of the NNRTI-based regimen's side effects. Almost 50 % of NNRTI efavirenz (EFV) users will have some kind of neuropsychiatric adverse event [66], contributing to discontinuation and LTFU [67,68]. It has been shown that EFV has a higher dropout rate than DTG. A pretreatment HIVDR study (2016) in Nepal also showed >10 % resistance to NNRTIs (nevirapine [NVP] and EFV) [69]. Based on similar findings, along with global recommendations, country-specific evidence [70], and updated Nepalese HIV treatment guidelines, a Dolutegravir (DTG)-based regimen (TLD: tenofovir, lamivudine, Dolutegravir) has been the preferred first-line treatment in Nepal since mid-2020.

Our study found that groups of highest risk of acquiring HIV also had the lowest likelihood of being retained in HIV care, consistent with previous research findings [71,72]. Factors for low retention in HIV care among sex workers have been reported such as internalized stigma related to sex work and sexual orientation, discrimination, sex work environment, criminalization of sex work, and negative attitudes towards HIV treatment [73,74]. In countries like Nepal where commercial sex is illegal, the criminalised status of their work means that commercial sex workers are prone to harassment and violence, have less knowledge about reproductive health, less control of whether or not they use condoms during sex and are less likely to take legal action against violence and abuse [75]. Similarly, PWID, MSM and TG also had higher LTFU incidence which is in line with previous studies [[76], [77], [78]]. MSM and TG women in Nepal experience a number of social and structural obstacles that have a substantial impact on their health and social position, including discrimination, lack of proper legal protection, diminished social capital, and mental health concerns [[79], [80], [81]]. Continuous prejudice and discrimination, particularly in households where family support is lacking, have also shown to be a contributing factor to the low uptake of HIV testing services among MSM and PWID in Nepal [78]. These conditions can, either alone or together, lead to barriers to access to health care, non-retention and ultimately poor health outcomes.

The results from this study should be interpreted considering several limitations. First, data for these analyses comes only from clients with complete information who were enrolled in ART clinics. Thus, LTFU among those never engaged in care or who had missing data were not included in this analysis. Second, we used secondary routine health facility data and were not able to examine factors outside these data that may have influenced LTFU among PLHIV in Nepal. Third, PLHIV in this study who were LTFU were not in communication with their health providers in the public health system. Some individuals classified as missing or LTFU in our dataset may have relocated to a different geographical area due to stigma and social rejection associated with HIV. In doing so, they may have re-registered under a different name at a new ART site and continued treatment there. This scenario was particularly plausible during periods when the majority of PLHIV were not yet enrolled in the biometric system. Alternatively, some of these clients may have discontinued treatment or died. Lastly, to preserve observations, we created an indicator for the education variable assuming that the missingness was at random and explained by inconsistent completion of medical records. However, this method may affect residual variance of the regression [82]. Despite minor limitations, a major strength is that the analysis was done using data from Nepal's national DHIS2 Tracker based healthcare database and is broadly generalizable to PLHIV on ART in Nepal. The utilization of programme data also gave us the advantage of describing and analyzing LTFU in a real-life setting of implementing test-and-treat. This study also demonstrates the application of routine health facility data collected through the open-source DHIS2 Tracker-based information system to monitor the effectiveness of interventions or track progress on key indicators prioritized by the national and global HIV strategies and targets.

## 5 Conclusion

In this nationwide sample of PLHIV, the risk of LTFU increased over time following the implementation of the universal test-and-treat strategy in Nepal. LTFU rates were higher among PLHIV who started ART within seven days of HIV diagnosis, younger individuals, those with lower education levels, Dalit, sex workers and their clients, PWID, MSM/TG, PLHIV at WHO stage 1, those on NNRTI regimens, and individuals from remote geographical areas.

In the era of test-and-treat and rapid ART initiation, it is crucial to regularly analyze context-specific facilitators and barriers to maximize the benefits of these interventions. In Nepal, the HIV treatment system and individual clients may not have been ready for the immediate enrollment into HIV care. Nepal may have been able to prevent LTFU with more time from a positive test result to HIV treatment enrollment by reducing the strain on the care system and giving clients time to process their new diagnosis and plan their futures. Adapting globally recommended strategies to the specific contexts of each country, geographical area, or subpopulation is essential. Otherwise, evidence suggests that highly efficacious interventions may fail to achieve the expected benefits when implemented in routine health programme due to health system failures or individual, family, and community-level factors.

## What this study adds

• This study found a moderately high incidence of loss to follow-up (LTFU) among people living with HIV (PLHIV) receiving early antiretroviral therapy (ART) in Nepal's universal test-and-treat setting, with an incidence rate of 4.22 per 100 person-years.
• It identifies key risk factors for LTFU, including early ART initiation (within seven days of diagnosis), younger age, being unmarried, Dalit and certain high-risk populations such as sex workers, men who have sex with men (MSM), and people who inject drugs (PWID).
• This study demonstrates that, despite the high effectiveness of the test-and-treat strategy in other settings, its anticipated benefits in improving retention may not be fully realized within the Nepalese healthcare system.

## Implications for policy and practice

• Targeted interventions are needed for high-risk groups identified as more likely to experience LTFU, including young people, unmarried individuals, Dalit, and key populations such as sex workers and PWID. This may involve tailored counseling, follow-up strategies, and enhanced youth-friendly services to improve retention.
• Policy adjustments should consider allowing a brief period for PLHIV to process their diagnosis and engage with support resources before ART initiation, as rapid initiation has shown associations with LTFU.
• Programs and interventions must adapt to ensure the test-and-treat strategy achieves its intended benefits in Nepal by addressing the unique needs of youth and certain key populations, managing early adverse drug reactions, and addressing systemic healthcare challenges to support effective implementation.

## Ethics approval and consent to participate

The Institutional Review Board of Nepal Health Research Council has approved the study. All participants provided informed consent prior to study participation. Permission was also obtained to digitally record all interviews. All procedures performed in studies involving human participants were in accordance with the ethical standards of the 2019 National Ethical Guidelines for Health Research in Nepal and with the 1964 Helsinki declaration.

## Authors’ contributions statement

AS and KD conceived and designed the study. KD, SB, RSK and LRP contributed to data curation. AS, LP, BA, and KD contributed to data analysis. AS, LP and KD drafted the first version of manuscript with inputs from BA, SB, RS, RSK, LRP, MBK, EW. All authors contributed substantially to manuscript drafting and revision, and approved submission of the manuscript.

## Funding

Funding for this study was provided by the national grant of The Global Fund to Fight AIDS, Tuberculosis and Malaria (GFATM). The funder had no role in study design, data collection and analysis, decision to publish, or preparation of the manuscript.

## Declaration of competing interest

The authors declare that they have no known competing financial interests or personal relationships that could have appeared to influence the work reported in this paper.
